# Effects of Dietary Phospholipids on Growth Performance, Digestive Enzymes Activity and Intestinal Health of Largemouth Bass (*Micropterus salmoides*) Larvae

**DOI:** 10.3389/fimmu.2021.827946

**Published:** 2022-01-11

**Authors:** Shilin Wang, Zhihao Han, Giovanni M. Turchini, Xiaoyuan Wang, Zishuo Fang, Naisong Chen, Ruitao Xie, Haitao Zhang, Songlin Li

**Affiliations:** ^1^Research Centre of the Ministry of Agriculture and Rural Affairs on Environmental Ecology and Fish Nutrition, Shanghai Ocean University, Shanghai, China; ^2^School of Life and Environmental Sciences, Deakin University, Geelong, VIC, Australia; ^3^Key Laboratory of Aquatic, Livestock and Poultry Feed Science and Technology in South China, Ministry of Agriculture and Rural Affairs, Zhanjiang, China

**Keywords:** phospholipids, larval fish, intestinal development, immune response, gut microbiota

## Abstract

While the beneficial roles of dietary phospholipids on health status and overall performances of fish larvae have been well demonstrated, the underlying mechanisms remain unclear. To address this gap, the present study was conducted to investigate the effects of dietary phospholipids on growth performance, intestinal development, immune response and microbiota of larval largemouth bass (*Micropterus salmoides*). Five isonitrogenous and isolipidic micro-diets were formulated to contain graded inclusion levels of phospholipids (1.69, 3.11, 5.23, 7.43 and 9.29%). Results showed that the supplementation of dietary phospholipids linearly improved the growth performance of largemouth bass larvae. The inclusion of dietary phospholipids increased the activity of digestive enzymes, such as lipase, trypsin and alkaline phosphatase, and promoted the expression of tight junction proteins including ZO-1, claudin-4 and claudin-5. Additionally, dietary phospholipids inclusion alleviated the accumulation of intestinal triacylglycerols, and further elevated the activity of lysozyme. Dietary phospholipids inhibited the transcription of some pro-inflammatory cytokines, including *il-1β*, and *tnf-α*, but promoted the expression of anti-inflammatory cytokines *tgf-β*, with these modifications being suggested to be mediated by the p38MAPK/Nf-κB pathway. The analysis of bacterial 16S rRNA V3-4 region indicated that the intestinal microbiota profile was significantly altered at the genus level with dietary phospholipids inclusion, including a decreased richness of pathogenic bacteria genera *Klebsiella* in larval intestine. In summary, it was showed that largemouth bass larvae have a specific requirement for dietary phospholipids, and this study provided novel insights on how dietary phospholipids supplementation contributes to improving the growth performance, digestive tract development and intestinal health.

## Introduction

Phospholipids are phosphorus containing lipids with several important structural and functional roles (1, 2). It is well demonstrated that the limited biosynthetic capacity in larval stages of most fish species makes phospholipids essential nutrients, and accordingly they have to be supplemented in larval diets (3). The provision of appropriate dietary phospholipids is beneficial in improving growth performance, digestive tract development, antioxidant capacity and stress resistance (4–7). However, there are still a series of unclarified physiological considerations and mechanisms that might contribute to the explanation of how dietary phospholipids affect digestive and intestinal development and health in larval fish.

In addition to limited phospholipid biosynthetic capacity, larval fish also possess an immature digestive tract, whereas well-developed and functioning digestive organs are essential to digest the feed and absorb its nutrients (Zambonino 8). Extensive literatures revealed that dietary nutrient composition directly influences the development of digestive tract in fish larvae (3, 9, 10). Notably, the maturation of digestive functions has been associated with dietary phospholipids content in several larval fish, such as European seabass (*Dicentrarchus labrax*) (4), pikeperch (*Sander lucioperca*) (5) and large yellow croaker (*Larmichthys crocea*) (6). Importantly, in all these studies the maturation of the digestive tract was assessed by measuring the activity of intestinal enzymes such as lipase, amylase, trypsin and alkaline phosphatase. However, within this context, little research attention has thus far been given to tight junctions. These are intercellular junctions, which are essential for epithelial adhesion and barrier function in various kind of tissues and organs, including the digestive system (11). The tight junctions have been suggested to regulate the intercellular structural integrity of intestine in some juvenile fish including gilthead seabream (*Sparus aurata*) (12), grass carp (*Ctenopharyngodon idella*) (13), and largemouth bass (*Micropterus salmoides*) (14). Nevertheless, little information is currently available on the influence of dietary phospholipids on tight junctions in fish larvae.

Phospholipids are important components of chylomicrons, which are central for lipid transport (15), but the enterocytes of larval fish possess limited phospholipid biosynthetic capability (16). Thus, a dietary phospholipid deficiency in fish larvae commonly results in excessive lipid deposition in intestinal enterocytes (17–19), which, in turn, is commonly associated with chronic inflammation (20, 21). The p38 mitogen-activated protein kinases (MAPK), a member of MAPK family, is involved in the regulation of several cellular processes, including inflammation (22). The nuclear factor kappa B (Nf-κB), a downstream target of the p38 MAPK signaling pathway, is a major signaling molecule in the regulation of cytokines production (23). Therefore, it has been suggested that, in teleosts, the p38 MAPK/Nf-κB pathway participates in the regulation of inflammation response (20, 24). However, it is yet unclear whether dietary phospholipids can alleviate the intestinal inflammation response in fish larvae through the p38 MAPK/Nf-κB pathway.

Another important consideration when it comes to digestive and intestinal health in larval fish is the intestinal microbiota (25). The diversity and abundance of intestinal microbiota influence a wide range of host biological processes such as digestion, mucosal system development, and disease resistance (26, 27). Growing evidence revealed that the dietary composition is affecting the intestinal microbiome in most fish species (28–30). Therefore, the possible beneficial roles of dietary phospholipids on the development of intestine in fish larvae may also be partly related to the variation in intestinal microbiota. However, little information is currently available on the influence of dietary phospholipids on intestinal microbiota.

Considering the points introduced above, and aiming at contributing to current knowledge of the roles of dietary phospholipids on the overall performance of fish larvae, the present study focused on assessing the effects of dietary phospholipids on growth performance, digestive enzymes activity, antioxidant capacity, immune response and intestinal microbiome of larval largemouth bass. Largemouth bass was chosen as target species as it has important socio-economic considerations, as its production in China has reached 600,000 tons in 2020 (31). Previous studies mainly focused on the nutritional physiology research of juvenile largemouth bass (32–36). However, on farm, the survival rate of this fish larvae is reported to be as little as 3%, which represents an important bottleneck for the further development of this aquaculture sector. As such, any knowledge gain in larval nutrition of this species would have dramatic positive effects on this industrial sector, as well as it would contribute to current fundamental knowledge of fish nutrition.

## Materials and Methods

### Experimental Diets

Five isonitrogenous, isoenergetic and isolipidic diets were formulated with graded supplementation of soybean phospholipids, 0% (control, PL0), 2% (PL2), 4% (PL4), 6% (PL6) and 8% (PL8), and the resulting measured value of dietary phospholipids content was 1.69, 3.11, 5.23, 7.43, and 9.29%, respectively (**Table 1**). Diets were manufactured following previously described procedures (36). Briefly, after thorough mixing of the dry ingredients, water was added to the mixture to make a stiff dough, which was then extruded through a 1.0 mm die by a pelleting machine, and dried in a ventilated oven at 55°C. Then, the experimental diets were ground to crumbles of four different particle sizes, 180-250 μm, 250-380 μm, 380-830 μm and above 830 μm, which were stored at -20°C until being used.

**Table 1:** Formulation and proximate composition of experimental diets (mg/g dry matter).

Ingredients	Diets				
	PL0	PL2	PL4	PL6	PL8
White fish meal^a^	580.0	580.0	580.0	580.0	580.0
Hydrolyzed fish paste^a^	80.0	80.0	80.0	80.0	80.0
Sodium caseinate^a^	40.0	40.0	40.0	40.0	40.0
Fermented soybean meal^a^	42.0	42.0	42.0	42.0	42.0
Blood meal^a^	20.0	20.0	20.0	20.0	20.0
Wheat gluten meal^a^	25.0	25.0	25.0	25.0	25.0
Shrimp meal^a^	40.0	40.0	40.0	40.0	40.0
Bread yeast hydrolysate^a^	5.00	5.00	5.00	5.00	5.00
Fish oil	15.0	15.0	15.0	15.0	15.0
Ca(H_2_PO_3_)_2_	10.0	10.0	10.0	10.0	10.0
Vitamin mixture^b^	13.0	13.0	13.0	13.0	13.0
Mineral mixture^c^	10.0	10.0	10.0	10.0	10.0
α-Starch^a^	40.0	40.0	40.0	40.0	40.0
Soybean phospholipids^a^	0.0	20.0	40.0	60.0	80.0
Soybean oil^a^	80.0	60.0	40.0	20.0	0.00
Proximate analysis (Mean values, mg/g dry weight)					
Crude protein	607.5	603.0	606.8	605.6	605.3
Crude lipid	139.5	137.4	133.3	133.0	136.9
Phospholipids	16.9	31.1	52.3	74.3	92.9

a Supplied by Xinxin Tian’en Aquatic Feed Co., Ltd (Zhejiang, China).

b Vitamin Premix (mg/kg diet): vitamin A, 16000 IU; vitamin D_3_, 8000 IU; vitamin K_3_, 14.72; vitamin B_1_, 17.80; vitamin B_2_, 48; vitamin B_6_, 29.52; vitamin B_12_, 0.24; vitamin E, 160; vitamin C, 800; niacinamide, 79.20; calcium-pantothenate, 73.60; folic acid, 6.40; biotin, 0.64; inositol, 320; choline chloride, 1500; L-carnitine, 100.

c Mineral Premix (mg/kg diet): Cu (CuSO_4_), 2.00; Zn (ZnSO_4_), 34.4; Mn (MnSO_4_), 6.20; Fe (FeSO_4_), 21.10; I (Ca (IO_3_)_2_), 1.63; Se (Na_2_SeO_3_), 0.18; Co (COCl_2_), 0.24; Mg (MgSO_4_·H_2_O), 52.70.

### Experimental Procedure

The *in vivo* feeding trail was conducted at the joint laboratory of Shanghai Ocean University and Guangdong Evergreen Feed Industry Co., Ltd (Zhanjiang, China), and all procedures of this study were performed following the guide for the use of experimental animals of Shanghai Ocean University. Largemouth bass larvae (7 DPH, days post hatching) were provided by a local commercial hatchery in Foshan city (Guangdong, China). Larvae were fed with newly hatched artemia for three days, and then weaned to adapt a dried commercial micro-diet by alternate feeding artemia and micro-diet over a six days’ period. Then, larvae (17 DPH) with initial body weight of 9.61 ± 0.02 mg were divided into 15 tanks (water volume 1000 L) with the destiny of 2500 individuals per tank. Following a random allocation, triplicates groups of larvae were fed the experimental diets to apparent satiation five times daily (6:00, 9:00, 12:00, 15:00, and 18:00) for 28 days. The larvae were gradually fed the diet with particle size of 180-250 μm (body weight ≤ 0.10g), 250-380 μm (0.10g < body weight ≤ 0.50g), 380-830 μm (0.50g < body weight ≤ 0.80g) and above 830 μm (body weight > 0.80g). The water quality was monitored during the feeding trail to maintain within optimal conditions: temperature, 27 ± 1°C; pH, 7.2 ± 0.2; dissolved oxygen ≥ 6 mg/L.

### Sampling

The initial body weight and body length of 100 randomly collected largemouth bass larvae were measured at the beginning of the feeding trail. At the termination of the *in vivo* study, the weight and number of fish larvae were recorded to calculate the survival rate and growth performance. Before final weighing, fish were fasted for 24h and then anaesthetized with eugenol (1:10000; Shanghai Reagent Corp., Shanghai, China). Thirty larvae were randomly collected and sacrificed to measure the individual body weight and body length, and were then used for the body composition analysis. Another sixty fish were sacrificed and dissected to collect liver and intestinal samples for the analysis of enzyme activity, gene expression and microbiome.

### Chemical Composition Analysis

The moisture content was determined through drying samples at 105°C to a constant weight (37). Crude protein content was measured with the Kjeldahl method through the measurement of nitrogen content (N × 6.25) (37). Crude lipid content was assayed by the chloroform-methanol extraction method (38) with some modification described in Peng etal. (39). Dietary phospholipids content was assayed with the molybdenum blue method following the procedure described in Li etal. (40).

### Digestive Enzymes Activity Analysis

The intestinal samples were homogenized in ice-cold phosphate buffer saline (1:9; w/v), and then centrifuged at 5000 rpm for 10 min at 4°C to separate the supernatant which was used for the analysis of digestive enzymes activity. The trypsin activity was measured by the trypsin assay kit (Nanjing Jiancheng Bio-Engineering Institute, China), and L-arginine ethyl ester was used as the reaction substrate. The activity of lipase was determined, with triglyceride as the substrate, by the lipase assay kit (Nanjing Jiancheng Bio-Engineering Institute). The amylase activity was assayed by evaluating the reduced amylase content with a commercial kit (Nanjing Jiancheng Bio-Engineering Institute). The activity of alkaline phosphatase was determined using disodium phenyl phosphate as reaction substrate with a commercial kit (Nanjing Jiancheng Bio-Engineering Institute). The soluble protein content was determined with the coomassie brilliant blue method (41), which content was measured to calculate the specific activity of digestive enzymes.

### Lysozyme Activity Analysis

The lysozyme activity of intestine was determined using the turbidimetric method with a commercial lysozyme assay kit (Nanjing Jiancheng Bio-engineering Institute). Briefly, the transmittance was recorded at 20s after samples being blended with the suspension of *Micrococcus lysodeikticus*. Then, the mixture was reacted at 37°C for 5 min, and thereafter, the transmittance was recorded to calculate the lysozyme activity.

### Triglyceride Content Analysis

The triacylglycerols (TAG) content in intestine was analyzed spectrophotochemically at 546 nm according to the colorimetric enzyme-linked TAG detection method described by Schwartz and Wolins (42) with a commercial TAG regent kit (Nanjing Jiancheng Bio-engineering Institute).

### RNA Extraction and Relative Gene Expression Analysis

The relative expression of genes involved in immune response and intestinal barrier function was determined following previously described procedures (36). The primer sequences for the selected target genes, *p38α mitogen-activated protein kinase* (*p38α mapk*/*mapk14*), *p38δ mapk* (*mapk 13*), *nuclear factor-kappa B p65* (*nf-κb p65*/*rela*), *interleukin-1β* (*il-1β*), *tumor necrosis factor-α* (*tnf-α*), *5-lipoxygenase* (*5-lox*), *transforming growth factor-β* (*tgf-β*), *zona occludens-1* (*zo-1*), *claudin-1*, *claudin-4*, *claudin-5* and *β-actin* were designed based on the corresponding sequences or referred to previously published studies (14, 43) (**Table 2**). The total RNA of whole intestine was extracted using RNAiso Plus (Takara, Japan), and then was reverse transcribed into single strand cDNA based on the description of Li et al. (21). The real-time quantitative PCR (RT-qPCR) was performed in a quantitative thermal cycler (CFX96, Bio-Rad, CA, USA) with the following procedure: 95°C, 2min; 40 cycles of 95°C for 10 s, 57°C for 10 s, and 72°C for 20s. After the reaction, a melting curve analysis was performed to confirm the single PCR product in those reactions. The relative expression of target genes was calculated using the 2^-ΔΔCt^ method (44), and *β-actin* was used as the housekeeping gene.

**Table 2:** Sequences of the specific primers used in real-time quantitative PCR.

Gene	Forward sequence (5’-3’)	Reverse sequence (5’-3’)
mapk13	CTGCTTGAGAAGATGCTGGTT	AGGCTGTCGAAATATGGGTG
mapk14	TTCGATGGAGACGAGATGG	GAGATGAATGACCGCAGGC
rela	GCTGGTGTCTGGTTCATT	GCCTCCTCTTCCATCTCT
il-1β	CGTGACTGACAGCAAAAAGAGG	GATGCCCAGAGCCACAGTTC
5-lox	GGGATTTTATCGGGGGAC	AACGAGGGAAAGAGGCTG
tgf-β1	GCTCAAAGAGAGCGAGGATG	TCCTCTACCATTCGCAATCC
tnf-α	CTTCGTCTACAGCCAGGCATCG	TTTGGCCACACCGACCTCACC
zo-1	ATCTCAGCAGGGATTCGACG	CTTTTGCGGTGGCGTTGG
claudin-1	CCAGGGAAGGGGAGCAATG	GCTCTTTGAACCAGTGCGAC
claudin-4	TAATCGCTATGGTGGGAGCC	GCCCCGATCTCCATCTTCTG
claudin-5	TGGGACTATCGATGTCGATA	CCACAAGCCGTCCCAGATAG
β-actin	CTCTGGGCAACGGAACCTCT	GTGCGTGACATCAAGGAGAAGC

mapk13, mitogen-activated protein kinase 13; mapk14, mitogen-activated protein kinase 14; rela, nuclear factor-kappa B p65; il-1β, interleukin-1β; 5-lox, 5-lipoxygenase; tgf-β1, transforming growth factor-β 1; tnf-α, tumor necrosis factor-α; zo-1, zona occludens-1.

### DNA Extraction, Amplification, and Sequencing of Gut 16S rRNA Genes

The bacterial DNA of the larvae in both the control group and PL8 group were extracted using the E.Z.N.A.^®^ soil DNA Kit (Omega, USA) following the instructions of manufacturer. The integrity of DNA extract was determined on 1% agarose gel, and concentration and purity of the obtained DNA were measured with NanoDrop 2000 UV-vis spectrophotometer (Thermo Scientific, Wilmington, USA). Then, the V3-V4 region of the bacterial 16S rRNA gene was amplified with the 338F/806R primer set in a PCR thermocycler (ABI, USA). The PCR program was set as follows: 95°C for 3 min, followed by 27 cycles of 95°C for 30 s, 55°C for 30 s and 72°C for 45 s, and 72°C for 10 min. Subsequently, the PCR product was extracted from 2% agarose gel, and purified with the AxyPrep DNA Gel Extraction Kit (Axygen, USA), and quantified using Quantus™ Fluorometer (Promega, USA). After that, the purified amplicons were sequenced on an Illumina MiSeq PE300 platform (Illumina, USA) by Majorbio Bio-Pharm Technology Co. Ltd. (Shanghai, China).

### Bioinformatic Analysis

The raw DNA sequences were demultiplexed and quality-filtered to obtain the high-quality clean reads with the Quantitative Insights into Microbial Ecology (QIIME) quality filters (45), and thereafter, merged with the FLASH software (46). Then, operational taxonomic units (OTUs) with a 97% similarity cutoff were clustered using UPARSE, and then chimeric sequences were removed UCHIME Algorithm (47). The phylogenetic affiliation of each OTU representative sequence was analyzed by RDP Classifier against the 16S rRNA database (eg. Silva v138), and a confidence threshold was set as 70% (48). The alpha diversity analysis, Shannon index, Simpson index, Ace index and Chao index, and beta diversity analysis, non-metric multidimensional scaling (NMDS) ordination, principal coordinates analysis (PCoA) and hierarchical clustering tree analysis, were calculated with QIIME and displayed with R software. Linear discriminant analysis (LDA) effect size (LefSe) analysis was used to identify the different abundant taxa between the two detected groups (49)

### Calculations and Statistical Analysis

The growth performance of largemouth bass in response to dietary phospholipids inclusion were calculated as follows:


Survival rate (SR, %)=final fish number/initial fish number×100;

Specific growth rate (SGR, %/d)=(Ln(final body weight)−Ln(initial body weight))×100/days.

All of the statistical analysis were performed with SPSS version 19.0 (SPSS, Inc.). Polynomial contrasts (linear and quadratic) were used to test the effects of dietary phospholipids inclusion levels on the various variables measured in the present study after the data being tested for normality and homoscedasticity. When both linear and quadratic significant regressions were observed, linearity was selected as the simplest model for describing the observed trend. The level of significance was set at *P* < 0.05, and the results are presented as mean values, plus or minus standard errors of means. The intestinal bacterial diversity and OTU richness were analyzed by the Welch’s t-test with statistically significance being considered as *P* < 0.05.

## Results

### Growth Performance

The supplementation of dietary phospholipids significantly increased the final body weight and final body length in a positive linear manner (*P* < 0.05) (**Table 3**). The increasing levels of dietary phospholipids led to a direct linear increase of the specific growth rate, with the larvae of PL8 group obtained the maximum value (*P* < 0.05) (**Table 3**). The survival rate also increased linearly with the increase of dietary phospholipids (*P* < 0.05), and the maximum content was observed in the PL8 group (**Table 3**).

**Table 3:** Growth performance of largemouth bass larvae fed the experimental diets for 28 days*.

	Diets					Pooled SEM	Regression (P/R^2^)	
	PL0	PL2	PL4	PL6	PL8		Linear	Quadratic
IBW (mg)	9.61	9.61	9.61	9.61	9.61	n.a.	n.a.	n.a.
FBW (g)	0.28	0.66	0.71	0.79	1.23	0.08	<0.001/0.832	<0.001/0.835
IBL (cm)	0.90	0.90	0.90	0.90	0.90	n.a.	n.a.	n.a.
FBL (cm)	2.32	2.93	3.02	3.42	3.82	0.14	<0.001/0.886	<0.001/0.887
SR (%)	9.95	14.63	14.14	19.74	21.04	1.11	<0.001/0.849	<0.001/0.850
SGR (%/day)	12.91	15.62	16.10	16.17	17.81	0.43	<0.001/0.768	<0.001/0.803

^*^Values are means of triplicate. SEM, standard error of means; n.a., not applicable; IBW, initial body weight; FBW, final body weight; IBL, initial body length; FBL, final body length; SR, survival rate; SGR, specific growth rate.

### Body Approximate Composition

The inclusion of phospholipids produced no significant influence on the moisture content of largemouth bass larvae (*P* > 0.05) (**Table 4**). However, the crude protein content was linearly related to the increase of dietary phospholipids (*P* < 0.05), and the maximum value was observed in the PL4 group (**Table 4**). The increase of dietary phospholipids led to a linear decrease of crude lipid content of whole fish body (*P* < 0.05) (**Table 4**).

**Table 4:** Effects of dietary phospholipids on whole body composition of largemouth bass larvae fed the experimental diets for 28 days*.

	Diets					Pooled SEM	Regression (P/R^2^)	
	PL0	PL2	PL4	PL6	PL8		Linear	Quadratic
Moisture (%)	79.11	77.01	77.70	77.51	77.32	0.25	n.s.	n.s.
Crude protein (%)	14.22	15.27	15.73	15.60	15.70	0.16	0.002/0.539	<0.001/0.796
Crude lipid (%)	4.32	5.00	4.05	3.78	4.00	0.12	0.017/0.366	n.s.

^*^Values are means of triplicate. SEM, standard error of means; n.s., no significant difference was observed.

### Digestive Enzyme Activities

The activity of amylase decreased as dietary phospholipids increased, although no linear, nor quadratic, response was observed (*P* > 0.05) (**Table 5**). However, the inclusion of dietary phospholipids led to a linear increase of lipase activity, and the maximum value was observed in PL8 group (*P* < 0.05) (**Table 5**). Meanwhile, the increase of dietary phospholipids linearly elevated the activity of trypsin increased with the maximum value being observed in the PL6 group (*P* < 0.05) (**Table 5**). The variation of alkaline phosphatase (AKP) followed a similar pattern with trypsin, and the maximum activity of AKP was achieved in the PL4 group (*P* < 0.05) (**Table 5**).

**Table 5:** The activities of digestive enzymes in the intestine of largemouth bass larvae fed with experimental diets for 28 days^*^.

	Diets					Pooled SEM	Regression (P/R^2^)	
	PL0	PL2	PL4	PL6	PL8		Linear	Quadratic
AMS (U/mgprot)	0.56	0.40	0.50	0.48	0.41	0.02	n.s.	n.s.
LPS (U/gprot)	24.31	36.77	36.57	24.19	42.30	2.26	0.014/0.380	0.044/0.406
Trypsin (U/mgprot)	887.64	1786.81	2572.52	2614.22	1808.80	185.16	0.046/0.273	<0.001/0.831
AKP (U/gprot)	93.26	177.55	218.92	179.34	181.99	12.08	0.047/0.270	0.001/0.694

^*^Values are means of triplicate. SEM, standard error of means; n.s., no significant difference was observed; AMS: amylase; LPS: lipase; AKP: alkaline phosphatase.

### Relative mRNA Levels of Tight Junction Proteins

**Figure 1:**
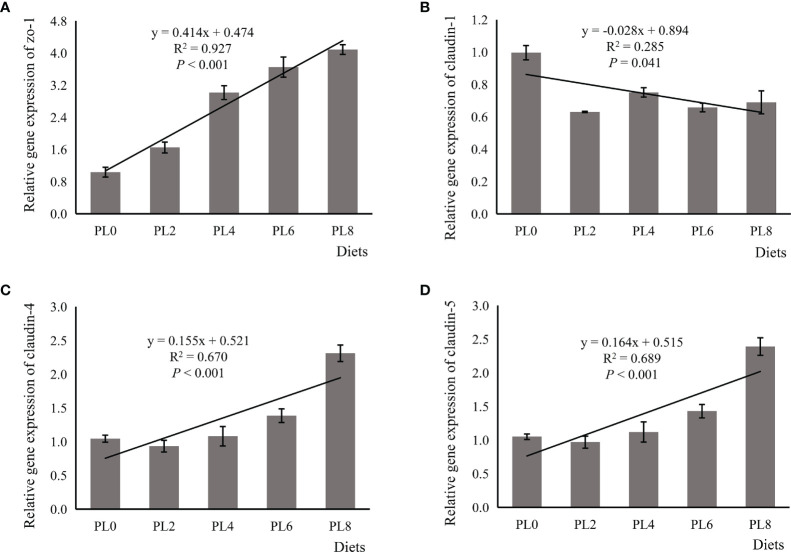
Relative expression of tight junction proteins, including zona occludens-1 (ZO-1) **(A)**, claudin-1 **(B)**, claudin-4 **(C)**, claudin-5 **(D)**, in the intestine of larval largemouth bass fed diets with grade levels of phospholipids for 28 days. ZO-1: *P*_linear_ < 0.001, $R_{\text{linear}}^{2}$ = 0.927; *P*_quadratic_ < 0.001, $R_{\text{quadratic}}^{2}$ = 0.952; claudin-1: *P*_linear_ = 0.041, $R_{\text{linear}}^{2}$ = 0.285; *P*_quadratic_ = 0.021, $R_{\text{quadratic}}^{2}$ = 0.474; claudin-4: *P*_linear_ < 0.001, $R_{\text{linear}}^{2}$ = 0.670; *P*_quadratic_ < 0.001, $R_{\text{quadratic}}^{2}$ = 0.900; claudin-5: *P*_linear_ < 0.001, $R_{\text{linear}}^{2}$ = 0.689; *P*_quadratic_ < 0.001, $R_{\text{quadratic}}^{2}$ = 0.902. The “x” in the regression equation means the variation of dietary phospholipids content.

The expression of *zo-1* was increased in linear manner as dietary phospholipids increased (*P* < 0.05) (**Figure 1**). The supplementation of phospholipids significantly decreased the expression of *claudin-1* in a negative linear manner (*P* < 0.05) (**Figure 1**). The expression of *claudin-4* and *claudin-5* was significantly followed an opposite pattern with that of *claudin-1*, and the lowest expression value was observed in larvae of the control group (*P* < 0.05) (**Figure 1**).

### Intestinal TAG Content, Lysozyme Activity and Immune Response

**Figure 2:**
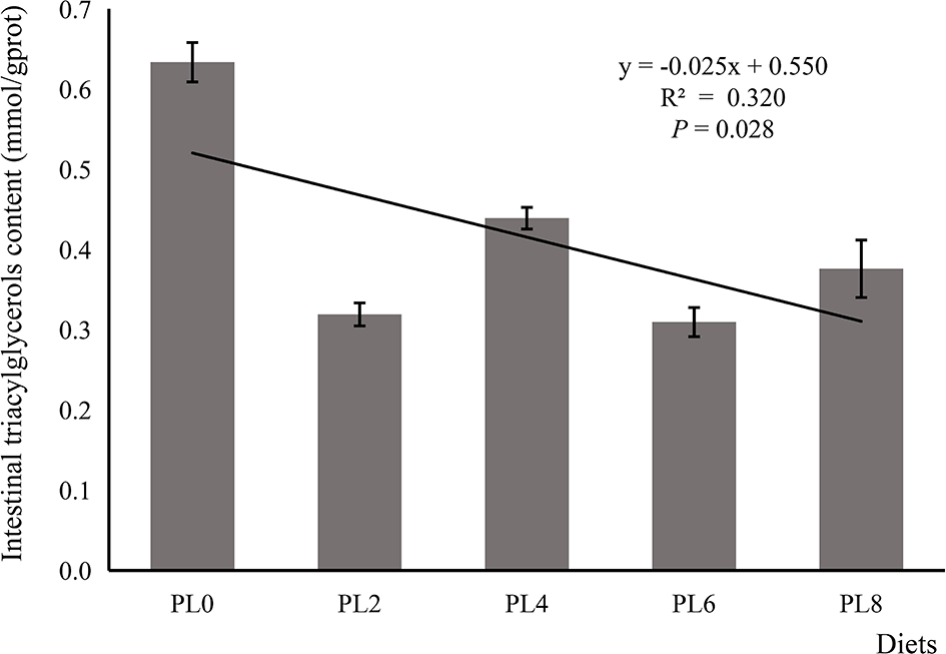
The triacylglycerols content in intestine of larval largemouth bass fed diets with graded levels of phospholipids for 28 days. TAG: *P*_linear_ = 0.028, $R_{\text{linear}}^{2}$ = 0.320; *P*_quadratic_ = 0.011, $R_{\text{quadratic}}^{2}$ = 0.525. The “x” in the regression equation means the variation of dietary phospholipids content.

**Figure 3:**
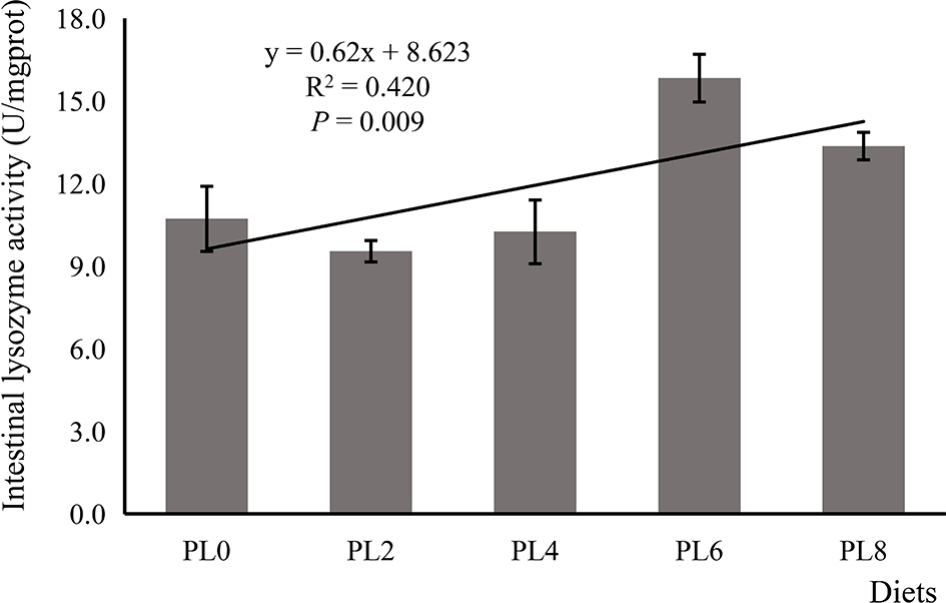
The lysozyme activity in intestine of larval largemouth bass fed diets with graded levels of phospholipids for 28 days. *P*_linear_ = 0.009, $R_{\text{linear}}^{2}$ = 0.420; *P*_quadratic_ = 0.037, $R_{\text{quadratic}}^{2}$ = 0.423. The “x” in the regression equation means the variation of dietary phospholipids content.

**Figure 4:**
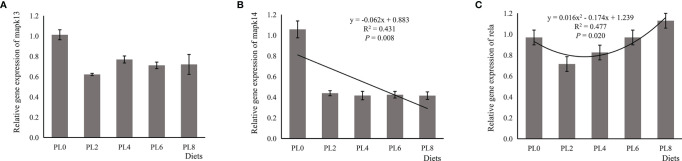
Relative expression of mitogen-activated protein kinase (MAPK) signal pathway related genes, including *mapk13* **(A)**, *MAPK14* **(B)**, and *rela* **(C)**, in intestine of larval largemouth bass fed diets with graded levels of phospholipids for 28 days. *mapk13*: *P*_linear_ = 0.202, $R_{\text{linear}}^{2}$ = 0.122; *P*_quadratic_ = 0.311, $R_{\text{quadratic}}^{2}$ = 0.177; *mapk14*: *P*_linear_ = 0.008, $R_{\text{linear}}^{2}$ = 0.431; *P*_quadratic_ < 0.001, $R_{\text{quadratic}}^{2}$ = 0.735; *rela*: *P*_linear_ = 0.881, $R_{\text{linear}}^{2}$ = 0.023; *P*_quadratic_ = 0.020, $R_{\text{quadratic}}^{2}$ = 0.477. The “x” in the regression equation means the variation of dietary phospholipids content.

**Figure 5:**
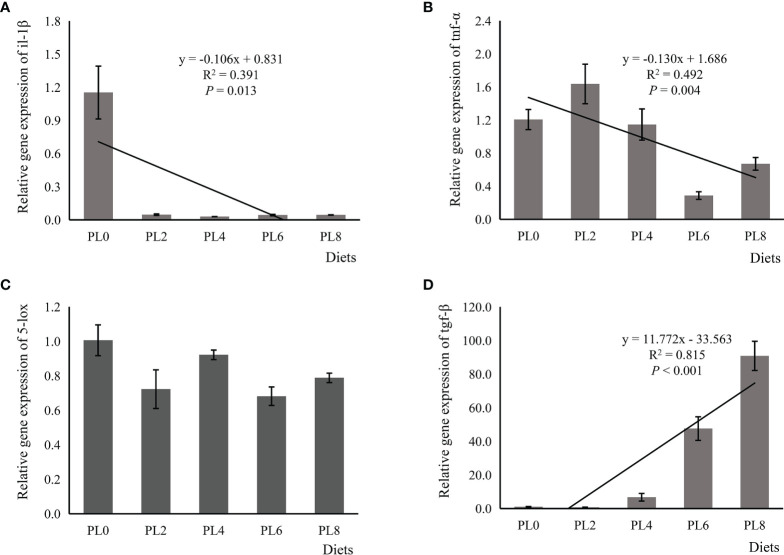
Relative expression of pro-inflammatory, including interleukin-1β (il-1β) **(A)**, tumor necrosis factor-α (tnf-α) **(B)**, and 5-lipoxygenase (5-lox) **(C)**, and anti-inflammatory transforming growth factor-β (tgf-β) **(D)** in intestine of larval largemouth bass fed diets with graded levels of phospholipids for 28 days. il-1β: *P*_linear_ = 0.013, $R_{\text{linear}}^{2}$ = 0.391; *P*_quadratic_ = 0.001, $R_{\text{quadratic}}^{2}$ = 0.695; tnf-α: *P*_linear_ = 0.004, $R_{\text{linear}}^{2}$ = 0.492; *P*_quadratic_ = 0.017, $R_{\text{quadratic}}^{2}$ = 0.493; 5-lox: *P*_linear_ = 0.132, $R_{\text{linear}}^{2}$ = 0.166; *P*_quadratic_ = 0.239, $R_{\text{quadratic}}^{2}$ = 0.212; tgf-β: *P*_linear_ = 0.000, $R_{\text{linear}}^{2}$ = 0.815; *P*_quadratic_ < 0.000, $R_{\text{quadratic}}^{2}$ = 0.956. The “x” in the regression equation means the variation of dietary phospholipids content.

The increased supplementation of dietary phospholipids showed a significant negative relationship with the TAG content in intestine of larval largemouth bass (*P* < 0.05) (**Figure 2**). The activity of lysozyme was increased in a linear manner with the increase of dietary phospholipids (*P* < 0.05) (**Figure 3**). The expression of *mapk14* was significantly down-regulated in a linear manner with the supplementation of phospholipids (*P* < 0.05) (**Figure 4**). Meanwhile, the inclusion of dietary phospholipids slightly decreased the expression of *mapk13* (*P* > 0.05) (**Figure 4**). The expression of *rela* was decreased quadratically with the increase of dietary phospholipids (*P* < 0.05) (**Figure 4**). The supplementation of dietary phospholipids led to linear down-regulation of *il-1β* expression (*P* < 0.05) (**Figure 5**). Meanwhile, the expression of *tnf-α* was also decreased linearly by dietary phospholipids (*P* < 0.05) (**Figure 5**). The inclusion of dietary phospholipids slightly depressed the expression of *5-lox*, although no significant regression was observed (*P* > 0.05) (**Figure 5**). The expression of *tgf-β* was linearly increased with the inclusion of dietary phospholipids (*P* < 0.05) (**Figure 5**).

### Microbiota Composition and Diversity Analysis

**Figure 6:**
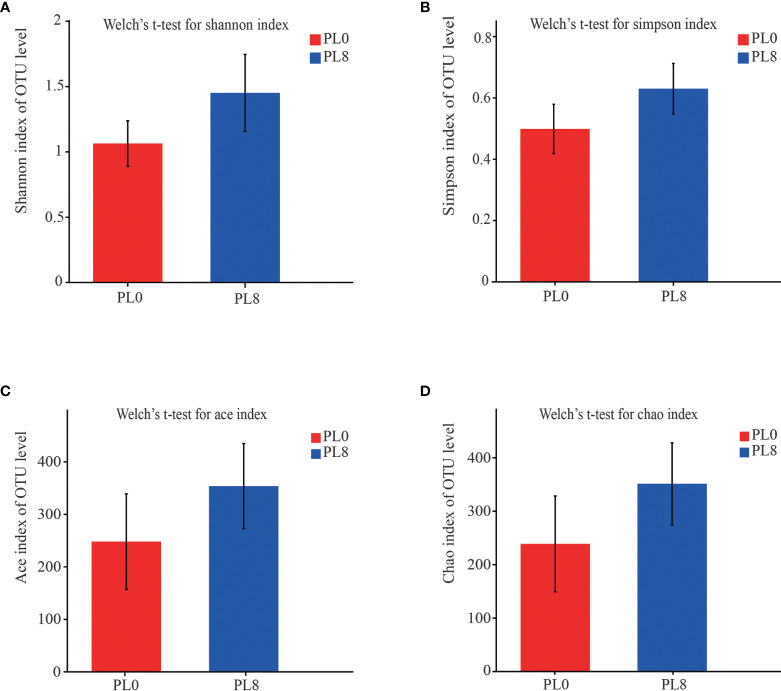
The alpha diversity comparisons analysis, including Shannon diversity index **(A)**, Simpson diversity index **(B)**, Ace species richness index **(C)** and Chao species richness index **(D)** of microbial communities in the intestine of largemouth bass in the PL0 (control) and PL8 groups. Values (mean ± standard error of the mean, SEM) in bars that have the same letter are not significantly different (*P* > 0.05; Welch’s t-test) between treatments (N = 3).

**Figure 7:**
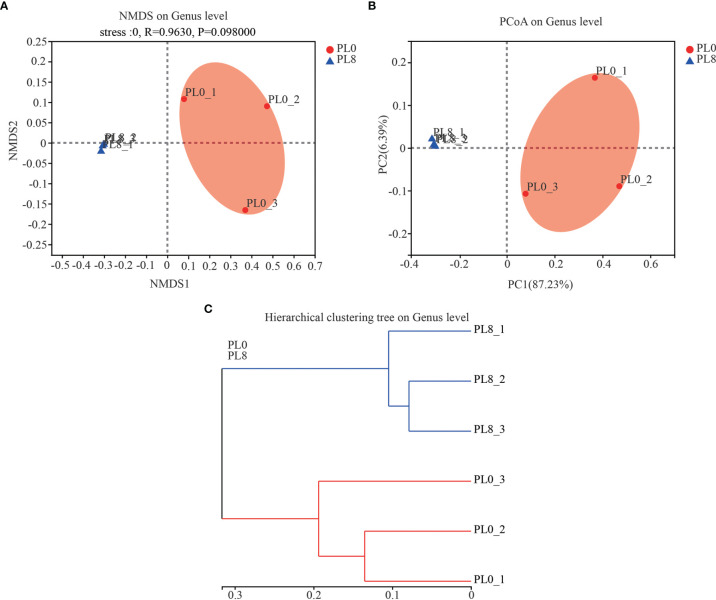
The beta diversity comparisons analysis, including non-metric multidimensional scaling (NMDS) **(A)**, principal component analysis (PCoA) **(B)**, and hierarchical clustering tree analysis **(C)** of microbial communities at genus level in the intestine of largemouth bass fed the diets in the PL0 (control) and PL8 groups.

A total number of 278,896 sequences were obtained after being demultiplexed, quality screened and trimmed, which contained 858 OTUs with 97% identity from 6 samples, and the raw reads were deposited into the NCBI Sequence Read Archive (SRA) database under accession number PRJNA767234. The OTUs were assigned to 638 species, 466 genera, 244 families, 136 orders, 54 class, and 26 phyla. The alpha diversity index, including Simpson, Shannon, Ace and Chao index, of the intestinal microbiota in larvae of PL8 group was slightly higher than that of the control group, but no significant differences were observed (**Figure 6**). The beta diversity index was estimated by principal coordinates analysis (PCoA) and non-metric multidimensional scaling (NMDS) ordination, and the results indicated that the intestinal bacterial in larvae of PL8 group was distinct from the control group (PL0) (**Figure 7**).

**Figure 8:**
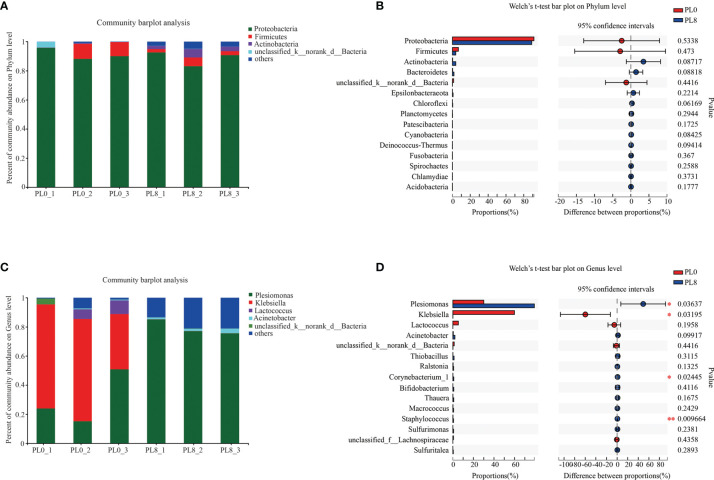
Relative abundances (%) of bacteria and comparison of bacterial abundances in the intestine of largemouth bass in the PL0 (control) and PL8 groups. at the phylum **(A, B)** and genus **(C, D)** level, and the phyla and genus with relative abundances lower than 1% were assigned as “others”, respectively. ^⁎^0.01 < P ≤ 0.05, ^⁎⁎^0.001 < P ≤ 0.01 (Welch’s t-test, N=3).

A bar map was used to graphically illustrate the variation in the dominant intestinal bacterial communities in response to dietary phospholipids at the phylum and genus level in PL0 (control) and PL8. The Proteobacteria, Firmicutes, and Actinomycetes were the dominant phyla in both treatments (**Figure 8**). However, *Plesiomonas*, *Klebsiella* and *Lactococcus* were detected as the predominant bacterial genera in the larval intestine of the control group, while *Plesiomonas* and *Acinetobacter* were the mainly intestine bacterial genera in the PL8 group (**Figure 8**). The Welch’s-test revealed that phospholipids supplementation significantly elevated the relative abundance of *Plesiomonas*, *Corynebacterium_1* and *Macrococcus*, but significantly decreased the abundance of *Klebsiella* (*P* < 0.05) (**Figure 8**).

**Figure 9:**
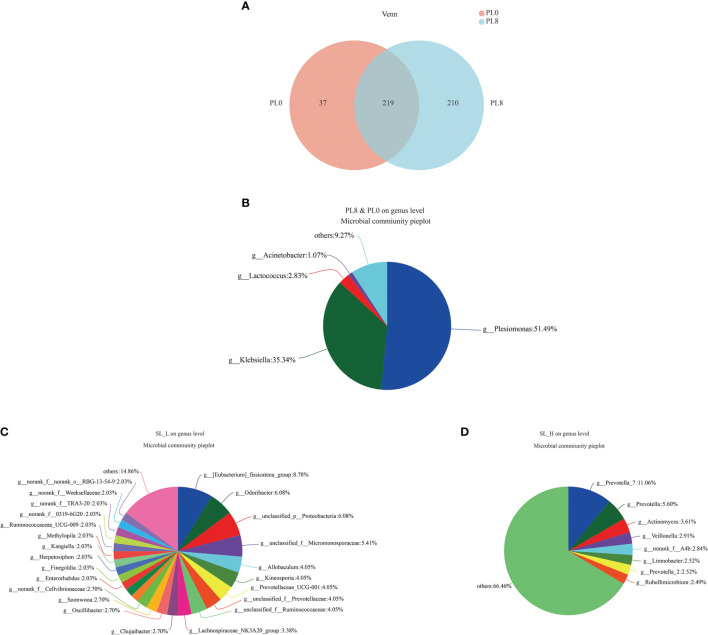
Venn diagram analysis of microbial communities in the intestine of largemouth bass in the PL0 (control) and PL8 groups. The number **(A)** and community **(B‐D)** of overlapping and unique bacterial genera **(C, D)** in the intestine of largemouth bass were identified.

The Venn diagram analysis revealed that 219 shared microbial genera were identified between the control group and PL8 group, and meanwhile, 37 and 210 unique bacterial genera were detected in the control group and PL8 group, respectively (**Figure 9**). The most abundant genera in the shared genera (> 10%) were *Plesiomonas* (51.49%) and *Klebsiella* (35.34%) (**Figure 9**). The unique intestinal microbial genus (>5%) in the larvae of the control group was *Eubacterium fissicatena*, *Odoribacter*, and genus unidentified *Proteobacteria* (**Figure 9**), and the unique genus (>5%) in the larvae of PL8 group was *Prevotella_7* and *Prevotella* (**Figure 9**).

**Figure 10:**
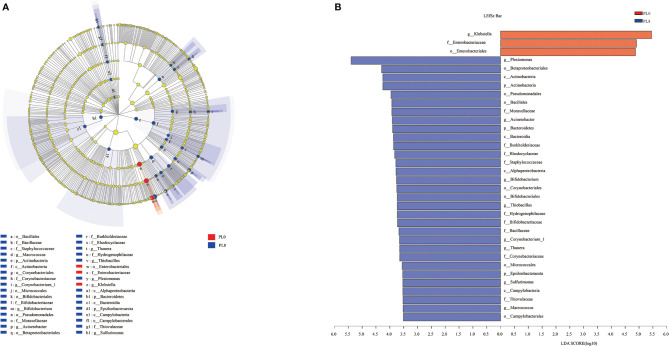
Cladogram showing the phylogenetic distribution of the bacterial lineages associated with dietary phospholipids inclusion. Taxonomic representation of statistically and biologically consistent differences between intestinal microbiota of largemouth bass in the PL0 (control) and PL8 groups **(A)**. Differences were represented by the color of the most abundant class (red indicates the control group; blue indicates PL8 group). Histogram of linear discriminant analysis (LDA) scores for differentially abundant taxon **(B)**. For interpretation of the references to color in this figure legend, the reader is referred to the web version of this article.

LEfSe analysis was conducted to evaluate the differences in intestinal microbial community composition from the domain to the genus level between the control group and PL8 group, and significant differences in taxonomic distribution of intestinal microbiota communities were observed (**Figure 10**). The larval intestine of the control group exhibited significant enrichment for order *Enterobacteriales*, family *Enterobacteriaceae* and genus *Klebsiella*, while high dietary phospholipids increased the relative abundance of *Sulfurimonas* (from phylum to genus), *Macrococcus*, Corynebacterium_1, *Bifidobacterium*, *Acinetobacter*, *Thauera*, and *Thiobacillus* (from order to genus) and *Plesiomonas* at the genus level (**Figure 10**).

## Discussion

It is well established that fish at larval stage possess a specific requirement for dietary phospholipids (1, 50). The improved growth performance caused by the inclusion of dietary phospholipids have been confirmed in some fish species, including European seabass, Pacific bluefin tuna (*Thunnus orientalis*), cobia (*Rachycentron canadum*), large yellow croaker, Dojo loach (*Misgurnus anguillicaudatus*), and hybrid grouper (*Epinephelus fuscoguttatus ♀*× *E. lanceolatus ♂*) (4, 6, 7, 51–53). Consistently, in the present study, the survival rate and specific growth rate were elevated with the supplementation of dietary phospholipids, and the maximum growth was observed in the PL8 group (9.29% phospholipids). As no plateau was observed with the increasing dietary supplementation of phospholipids, it can be concluded that the minimal requirement of phospholipids for optimal growth in larval largemouth bass is at least 9.29%, but possibly further improved performances could be achieved by higher supplementation levels. However, the findings of some previous studies revealed the dietary phospholipids requirement of larval freshwater fish (2-5%) was relatively low, and typically lower than marine fish species (5-12%) (1). Therefore, the relatively high phospholipids requirement observed here for the freshwater carnivorous fish largemouth bass seems to be a species-specific peculiarity, placing this species amongst marine larvae in terms of dietary phospholipids requirements. Prior to the feeding trail, the larvae were weaned with newly hatched artemia containing relative low phospholipids, about 4%, which was possibly far from meeting the phospholipids requirement of larval largemouth bass. Thus, it cannot be excluded that the nutritional background of experimental fish may have also affected the requirement of largemouth bass larvae for phospholipids observed in this experiment.

The immature digestive tract of fish larvae seriously restricts the digestion and absorption of nutrients, and that is one of the main reasons why the culture of larval fish relies on live prey. Previous studies have demonstrated that dietary phospholipids supplementation can promote the development of digestive tract in some fish larvae (4–6). In general, a decreased amylose activity and increased alkaline phosphatase indicate the maturation of the digestive tract (4). Consistently, in the present study, the supplementation of phospholipids slightly decreased the activity of amylose, and meanwhile a significantly elevated alkaline phosphatase activity was recorded. Additionally, the activity of lipase and trypsin were linearly elevated with the supplementation of dietary phospholipids, and this was consistent with the increased lipase and trypsin activity that were also observed in some previous studies in the same species (6, 54). In summary, the variation in digestive enzyme activity confirmed the beneficial roles of dietary phospholipids in improving digestive tract development in largemouth bass larvae.

Tight junctions are key to epithelial adhesion and barrier function in mammals (11). The tight junction protein ZO-1 acts as a bridge between the transmembrane protein occludin and cytoskeleton proteins (55). The downregulation of *zo-1* expression caused the impairment of tight junction (56) and, in teleosts, was associated with the disrupted intestinal structural integrity (14, 57). In addition, the activation of claudin 1, claudin 4 and claudin 5 are suggested to strengthen the junction of epithelial cells (58, 59). In the present study, the inclusion of dietary phospholipids was shown to directly up-regulate the expression of *zo-1*, *claudin 4* and *claudin 5*, which confirmed the positive regulation of dietary phospholipids on intestinal structural integrity. Interestingly, the intercellular intestinal structural integrity has been reported to be inversely related to the expression of *claudin 1* in largemouth bass (14), which was the opposite of what has been reported for mammals. Consistently, in the present study, the expression of *claudin 1* was decreased in a linear manner with the inclusion of dietary phospholipids, and therefore favored the development of intestinal integrity. All these results observed on the digestive enzyme activities and the tight junction protein expression confirmed the beneficial roles of dietary phospholipids in the physiological and healthy development of the digestive tract of fish larvae.

An insufficient supply of dietary phospholipids has been reported to generally promote lipid accumulation in the intestine of fish larvae, further resulting in intestinal histological damage (18, 19). This was suggested to be related to the limited phospholipids biosynthesis ability (16). In the present study, the supplementation of dietary phospholipids linearly decreased the TAG content in the intestine, with similar observation in larval gilthead sea bream (17) and common carp (18). This excessive lipid accumulation has been suggested to cause chronic inflammation in teleosts (20, 36). The immune barrier function, mainly depending on antibacterial compounds, such as lysozyme and cytokines, is also an important component of intestinal mucosal barriers (60). In the present study, the supplementation of dietary phospholipids proportionally increased the activity of intestinal lysozyme of largemouth bass. The increased lysozyme activity induced by dietary phospholipids inclusion has been observed in some fish species, such as Caspian brown trout (*Salmo trutta caspius*) (61), common carp (*Cyprinus carpio*) (62) and stellate sturgeon (*Acipenser stellatus*) (63). Cytokines, including pro-inflammatory and anti-inflammatory cytokines, are mainly responsible for the host innate defense in fish (64). The pro-inflammatory cytokine *tnf-α* is an important mediator in the regulation of inflammatory response, and its activation induces gene expression of some proinflammatory factors, such as *il-1β*, in rainbow trout (65). In the present study, the expression of pro-inflammatory cytokines, *tnf-α* and *il-1β*, were repressed linearly with the increased supplementation of dietary phospholipids. The anti-inflammatory cytokines, such as *tgf-β*, can depress the production of pro-inflammatory cytokine, and thereby inhibit the inflammatory response in teleost (66). In general, the expression of anti-inflammatory cytokines commonly followed an opposite pattern with that of pro-inflammatory cytokines, which was previously observed in some other fish species (67, 68; 69). Consistently, the inclusion of dietary phospholipids was directly relative to the increase of the expression of *tgf-β*. Therefore, the elevated lysozyme activity, reduced expression of pro-inflammatory cytokines, and improved gene expression of anti-inflammatory cytokines confirmed the contribution of dietary phospholipids in improving fish immune response.

In mammals, the pro-inflammatory cytokines, such as *tnf-α* and *il-1β*, can be directly induced by the transcriptional factor, *nf-κb* (70). Previous studies have confirmed that the inhibition of *rela*, a member of *nf-κb* family with the function in regulating the transcription of target genes (71), decreased the expression of proinflammatory cytokines (72, 73). The positive relationship between the expression of *rela* and pro-inflammatory cytokines, such as *tnf-α* and *il-1β*, were widely observed in some fish species, including grass crap (67), large yellow croaker (68) and hybrid grouper (69). In the present study, the inclusion of dietary phospholipids significantly decreased the expression of *rela* in a quadratic manner, followed a similar pattern with that of the proinflammatory cytokines. Moreover, *nf-κb* is a downstream target of the p38 MAPK signaling pathway, which is involved in the regulation of inflammation in mammals (22) and is suggested to influence the intestinal immune function of grass carp (24, 74). In the present study, the transcription of *p38 mapk*, *mapk* 13 and *mapk* 14, was repressed with the inclusion of dietary phospholipids. The above results suggested that dietary phospholipids supplementation attenuated the inflammation response through the p38 MAPK/NF-κB pathway.

The assembly of intestinal microbiota into distinct communities are important during the development of fish larvae (75). It is well demonstrated that dietary nutritional composition can affect the intestinal microbiota composition and diversity in fish species (28, 76, 77). In the present study, dietary phospholipids supplementation produced no significant difference in the intestinal microbial community composition at the phyla level, and the Proteobacteria, Firmicutes, and *Actinomycetes* were the dominant phyla in both the two extreme treatments with the supplementation of phospholipids (PL8) or not (PL0, control), in accordance with previous studies reviewed by Borges etal. (78). However, the intestinal bacterial diversity was significantly affected at the genus level as dietary phospholipids increased. It’s well confirmed that the intestinal microbiota plays an important role in the regulation of immune function and protection against pathogens (79). The inclusion of dietary phospholipids significantly decreased the richness of genera *Klebsiella*, an enterobacterium that was suggested to cause inflammation response through the p38 MAPK pathway (80, 81). The variation in the intestinal microbiota observed in the present study confirmed the beneficial role of dietary phospholipids in attenuating the inflammation response of largemouth bass larvae, and provided novel insights on possible mechanisms.

In conclusion, this study showed that larval largemouth bass have a specific requirement for phospholipids, that this requirement is equal or greater than 9.29%, a requirement that is substantially higher compared to other freshwater species, and that the supplementation of dietary phospholipids significantly improved the growth performance and survival rate of the larvae. In addition, dietary phospholipids inclusion significantly improved the intestinal development, reduced inflammation response and modulated intestinal microbiota, which contributed to the beneficial roles of dietary phospholipids on the overall performance of largemouth bass larvae.

## Data Availability Statement

The original contributions presented in the study are publicly available. This data can be found here: National Center for Biotechnology Information (NCBI) BioProject database under accession number PRJNA767234.

## Ethics Statement

The animal study was reviewed and approved by the Animal Care and Use Committee of the Shanghai Ocean University.

## Author Contributions

SW: investigation, formal analysis, and writing - original draft. ZH: investigation, methodology, and data curation. GT: methodology and writing - review & editing. XW: investigation and methodology. ZF: investigation and methodology. NC: conceptualization, project administration, and funding acquisition. RX and HZ: investigation. SL: conceptualization, supervision, writing - review & editing, and funding acquisition. All authors contributed to the article and approved the submitted version.

## Funding

This work was financially supported by National Key R&D Program of China (2019YFD0900203), China Agriculture Research System of MOF and MARA (CARS-46), Shanghai Talent Development Fund (2019097), and National Natural Science Foundation of China (31802308).

## Conflict of Interest

The authors declare that the research was conducted in the absence of any commercial or financial relationships that could be construed as a potential conflict of interest.

## Publisher’s Note

All claims expressed in this article are solely those of the authors and do not necessarily represent those of their affiliated organizations, or those of the publisher, the editors and the reviewers. Any product that may be evaluated in this article, or claim that may be made by its manufacturer, is not guaranteed or endorsed by the publisher.
